# Process Optimization of Fluidized Bed Drying for Water Spinach: Evaluating the Effect of Blanching Through RSM and ANN Models

**DOI:** 10.1002/fsn3.70114

**Published:** 2025-03-24

**Authors:** Mir Tuhin Billah, Noor E Zannat, Md Akram Hossain, Ishmam Haque Sachcha, Sabina Yasmin, Md. Sazzat Hossain Sarker

**Affiliations:** ^1^ Department of Food Engineering and Technology, Faculty of Engineering Hajee Mohammad Danesh Science and Technology University (HSTU) Dinajpur Bangladesh; ^2^ Department of Computer Science and Engineering American International University‐Bangladesh Dhaka Bangladesh; ^3^ Department of Food Processing and Preservation, Faculty of Engineering Hajee Mohammad Danesh Science and Technology University (HSTU) Dinajpur Bangladesh

**Keywords:** artificial neural network (ANN), fluidized bed drying, optimization, predictive modeling, response surface methodology (RSM), water spinach

## Abstract

The quality of the dried leafy vegetables, such as water spinach (*Ipomoea aquatica*), has been found to be significantly affected by the drying process in terms of moisture content and retention of important nutrients, namely vitamin C and β‐carotene. There is great potential for fluidized bed drying to be applied for leafy vegetables in optimizing process parameters for maximum nutrient retention since it has not been researched. This work investigated the effect of temperature, drying time, and bed thickness on the nutritional quality of blanched and unblanched water spinach samples. In the present study, the fluidized bed drying process has been designed and optimized using a Central Composite Design (CCD) and Response Surface Methodology (RSM). For this study, both RSM and artificial neural network (ANN) predictive models are developed for further comparison. Using a multiobjective desirability function, the best‐optimized response was given from the experimental model for the responses of moisture content, vitamin C, and β‐carotene retention. Appropriate statistical metrics are applied, for example, AARD (Absolute Average Relative Deviation), MRD (Mean Relative Deviation), MSE (Mean Squared Error), and *R* ^2^ (Coefficient of Determination), which helped in model comparison during the study. It was observed from the experiment that all the response variables are significantly affected by drying temperature, time, and bed thickness. Variation of bed thickness in the blanched samples affected > 16% in moisture content attainment compared to unblanched samples, and vitamin C content exhibited a variation of more than 25% due to changes in bed thickness for blanched samples on the contrary. RSM has shown a better performance than ANN in its precision and prediction power. The optimized drying conditions came out to be 60°C as the drying temperature, 7.19 min as the drying time, and 5.12 cm as the bed thickness, which resulted in 2.95% of moisture content, 5.99 mg/100 g vitamin C, and 139.16 μg/g of β‐carotene. The close alignment between predicted and experimental values confirms the suitability of the optimized conditions for industrial‐scale drying of leafy vegetables.

## Graphical abstract

Fluidized bed drying optimizes vitamin C and β‐carotene retention in water spinach. Optimal drying: 60°C, 7.19 min, 5.12 cm bed thickness, achieving nutrient‐rich results. RSM outperforms ANN to predict responses during the drying process, predicted and experimental values align closely encouraging large‐scale application.

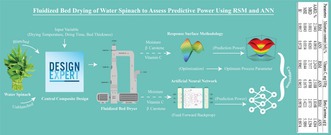

## 1 Introduction

Water spinach (*Ipomoea aquatica*) is considered to be one of the most nutritionally dense green vegetables consumed in Bangladesh, particularly because of its contents of vitamins A and C, beta‐carotene, and some essential minerals (Sarkar et al. 2023). Its ease of cultivation and low procurement cost make water spinach very common in local diets. Its nutrient density, especially due to its beta‐carotene content—a provitamin A—underlines its importance in the diet for the prevention of chronic diseases like stroke and heart disease (Kritchevsky et al. 1998; Pinilla‐González et al. 2024). For that reason, it is not only eaten fresh, but there are also prospects for water spinach use in dried form as a fortifying agent in various foodstuffs (Kozioł et al. 2024; Kruger et al. 2020).

However, this has to be done without loss of nutritional value during drying. Traditional techniques like sun drying, freeze drying, reactance window drying, pulse electric field (PEF), microwave drying, and cabinet drying have been tried for a number of leafy vegetables like spinach; however, all these methods usually result in substantial loss of nutrients (An‐Erl King et al. 2001; Galla et al. 2017; Grace et al. 2022; Ozkan et al. 2007; Syamila et al. 2019; Vargas et al. 2022; Yadav and Sehgal 1995; Yamakage et al. 2021). The fluidized bed dryer, being one of the more advanced drying technologies available, becomes a very promising alternative because it manages to maintain high heat and mass transfer rates while guaranteeing equable moisture reduction and shorter drying times. This technology, typically applied to the drying of granular agricultural products like rice, turned out to have potential in preserving quality in the dried product.

Despite the advantages of fluidized bed drying, little attention has been given to its application on water spinach. It has been previously applied to a variety of agricultural products like pepper, pearl millets, maize grain, carrots, and pistachio nuts, which demonstrates its great flexibility in the treatment of a wide array of plant materials and products (Chuwattanakul et al. 2022; Chuwattanakul and Eiamsa‐ard 2019; Mondal et al. 2021; Nazghelichi et al. 2010; Özahi and Demir 2015; Yogendrasasidhar and Setty 2019). Effects of fluidized bed drying on the quality of water spinach remain yet to be answered, especially on the retention of nutrients. Moreover, the critical drying parameters such as temperature, time, and bed thickness have not been properly researched for their effects on the dried water spinach.

Water spinach is nutritionally important; hence, the optimization of the drying method for maximum retention of its nutrients is quite important. Fluidized bed drying, with its inherent advantages, could perhaps be more effective than the conventional methods of drying (Sarker et al. 2015). However, in order to really use such drying technique's full potential, optimization of the drying parameters becomes imperative in order to understand their effects on final product quality. Moreover, though Response Surface Methodology (RSM) has been applied as an alcoholic in process optimization in the studies related to drying and extraction, Artificial Neural Network (ANN) represents the modern alternative solution that adds the ability for modeling complex and nonlinear relations among process variables and responses (Chokphoemphun et al. 2024; Hossain et al. 2024; Nanvakenari et al. 2021). Comparing these two predictive models would bring more insight and result in more accurate optimization, finally leading to the preservation of the nutritional integrity of water spinach during drying. Therefore, the objectives of the study were to assess the suitability of fluidized bed drying for water spinach leaves and to optimize the drying process; and to compare the prediction power of RSM and ANN based on the retention responses.

In the current study, it is attempted to associate the knowledge gap by probing the fluidized bed dryer (FBD) for drying water spinach. To enhance the precision of our study, a Central Composite Design (CCD) and RSM were used in optimizing the drying process. In our study, the next tool employed was an artificial neural network for modeling drying behavior and estimating moisture content, the amount of vitamin C, and beta‐carotene. Therefore, by comparing RSM and ANN on their performances in the prediction of these parameters, the study is able to establish the most effective drying conditions that preserve the nutritional quality of water spinach.

## 2 Materials and Methods

### 2.1 References of Unit Conversion

**Table:** 

Units used in the research	SI unit	Conversion factor
Gram, g	Kilogram, kg	1 g = 10^−3^ kg
Milligram, mg	Kilogram, kg	1 mg = 10^−6^ kg
Micro gram, μg	Kilogram, kg	1 mg = 10^−9^ kg
Centimeter, cm	Meter, m	1 cm = 10^−2^ m
Nanometer, nm	Meter, m	1 nm = 10^−9^ m
Milliliter, mL	Liter, L	1 mL = 10^−3^ L
Minute, min	second, s	1 min = 60 s

### 2.2 Material Collection and Sample Preparation

Fresh and mature water spinach ( *Ipomoea aquatica* ) leaves were procured from a local market in Dinajpur, Bangladesh. The leaves were harvested within a two‐week period from the onset of leaf emergence. Upon collection, the water spinach leaves were transported to the Food Engineering and Technology (FET) laboratory at Hajee Mohammad Danesh Science and Technology University (HSTU), Dinajpur. The leaves were thoroughly washed in potable water to remove any adhering dirt or debris. Subsequently, the petioles were removed, and the remaining leaf material was allowed to drain at ambient temperature.

Two sets of samples were prepared: blanched and unblanched. For blanching, 50 g of clean water spinach leaves were immersed in 250 mL of hot water (65°C–70°C) in a covered saucepan. The leaves were blanched for 3 min using an electric oven. Post‐blanching, the leaves were drained on a wire mesh to remove excess moisture. Due to their higher moisture content, blanched samples were unsuitable for direct fluidized bed drying. Therefore, these samples were subjected to sun drying for 45 min to reduce their moisture content to a level compatible with fluidized bed drying.

The unblanched samples, which had a lower initial moisture content, were directly loaded into the FBD after draining.

### 2.3 Overall Methodology

**FIGURE 1:**
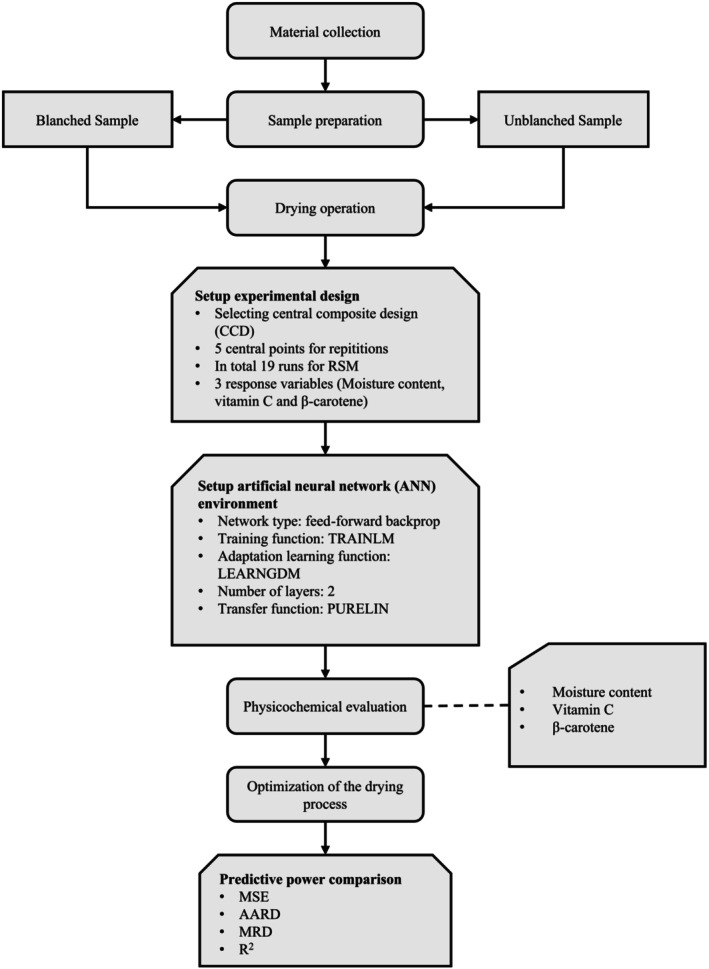
Diagram of the overall research approach.

The studies were carried out at Hajee Mohammad Danesh Science and Technology University, Dinajpur, Bangladesh. The main steps of the experimental procedure are presented below (Figure 1). Collecting the leaves of water spinach and arranging for drying: two categories of samples were taken, which include blanched and unblanched samples.

The central composite design was employed within a response surface methodology with the help of Design Expert software to generate experimental conditions. The experimental conditions were also established using the same conditions in an artificial neural network environment.

Determination of the target responses, namely moisture content, vitamin C, and beta‐carotene, was made using established analytical methods. The predictive performance of the RSM and ANN was compared with the results obtained from experimental runs. The RSM approach was used to determine the best drying conditions before subjecting water spinach leaves to optimized drying conditions in the FBD for the preparation of dried powder.

### 2.4 Drying Operation

**FIGURE 2:**
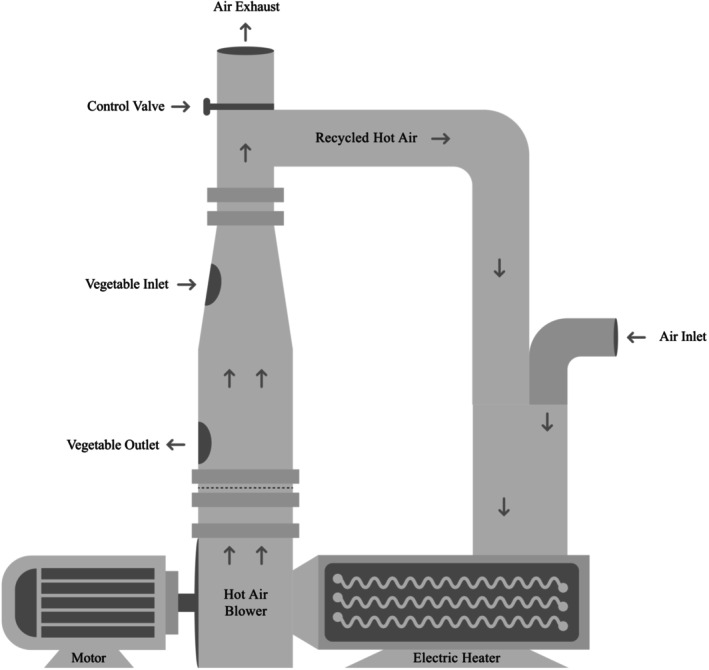
Schematic diagram of the lab‐scale fluidized bed dryer (FBD); original diagram by the authors, information based on (Akhtaruzzaman et al. 2021).

A laboratory‐scale batch‐type FBD developed earlier at the Department of FET, HSTU, Dinajpur, was used for the drying experiments. Figure 2 shows that it had a cylindrical drying chamber measuring 1.0 m high and 0.2 m in diameter with an air distributor to maintain uniform airflow through the chamber. An electric heater, rated at 18.0 kW, was used to increase the drying temperature above the surrounding atmosphere. A backward curve blade centrifugal blower, powered by a 2.2 kW electric motor operating at 2900 rpm, was utilized to provide the fluidizing air. In the current study, a constant bed air velocity of 4.5 m/s^−1^ was maintained throughout the drying process. A control system based on an automatic temperature controller was used to regulate and control the drying temperature as per the design expert running conditions. During drying, part of the exhaust air was recirculated in order to recycle some of the energy (Akhtaruzzaman et al. 2021).

### 2.5 Experimental Design Setup

The design of experiments (DOE) is a powerful method utilized to assess the effects of multiple independent variables and their interactions on the response‐dependent variables, thereby significantly reducing the number of experiments required in a process (Asfaram et al. 2018; Nanvakenari et al. 2021). Response Surface Methodology (RSM) has been highly recommended as a statistical approach, mostly when assessing optimization studies and food production. It allows for modeling the experimental data and exploring the effects of quantitative and qualitative independent variables on the output parameters, which fosters the identification of optimal conditions to secure desired responses with the minimum highly reliable number of tests possible (Majdi et al. 2019). ANOVA can also be used for data screening and to assess the reliability of the identified models, more particularly by considering the lack‐of‐fit values (Movagharnejad et al. 2019).

In this study, therefore, the main independent numerical variables under which the effects on output variables, like moisture content, quantity of vitamin C retention, and β‐carotene, are evaluated are the drying temperature, drying time, and bed thickness by employing the RSM. The experimental design was based on the CCD, which is generally considered the most common design and suitable for sequential experimentation. It also requires sufficient data to assess the lack of fit without requiring an inordinately large number of design points (Marget and Morris 2019). The parameters of the fluidized bed dryer to be varied were temperature (50°C–60°C), time (6–10 min), and bed thickness (3–6 cm). Experimental conditions and their corresponding levels for the applied response surface CCD in this work are represented in Table 1. The trials were carried out independently for blanched as well as for unblanched samples.

**TABLE 1:** Independent variable values of the process and their corresponding ranges and levels.

Name of input parameters	Notation symbol in equation	Range and levels		
		−1	0	+1
Drying temperature (DT), °C	A	50.00	55.00	60.00
Drying time (Dt), min	B	6.00	8.00	10.00
Bed thickness (Bt), cm	C	3.00	4.50	6.00

The Design Expert 12 software (trial version) was used to carry out a detailed analysis of the influence of independent variables, data processing, and optimization of the process. Table 2 shows the designed experiments and the corresponding results.

**TABLE 2:** Central composite design for the experimental variables of the FBD process and results.

Run no.	Experimental design			Results of samples without blanching			Results of samples with blanching		
	Drying temperature, °C	Drying time, min	Bed thickness, cm	Moisture content (wb), %	Vitamin C, mg/100 g	Beta‐carotene, μg/g	Moisture content (wb), %	Vitamin C retention, mg/100 g	Beta‐carotene, μg/g
1	60.00	6.00	3.00	2.04	4.10	96.26	2.43	3.09	106.93
2	63.00	8.00	4.50	2.18	4.40	103.00	2.60	3.30	114.50
3	55.00	8.00	4.50	2.50	5.10	118.40	3.00	3.79	131.23
4	55.00	8.00	4.50	2.40	5.30	127.90	3.10	3.65	140.00
5	50.00	6.00	6.00	4.89	10.00	230.00	5.90	7.41	255.00
6	50.00	10.00	3.00	1.47	3.00	70.25	1.80	2.22	77.00
7	50.00	10.00	6.00	2.93	6.00	135.00	3.50	4.45	154.00
8	60.00	6.00	6.00	4.07	8.20	194.50	4.90	6.18	212.80
9	60.00	10.00	6.00	2.44	4.95	119.00	2.95	3.71	127.50
10	55.00	11.40	4.50	1.76	3.60	84.89	2.10	2.66	93.10
11	55.00	8.00	2.00	1.09	2.25	54.90	1.30	1.67	58.50
12	55.00	4.60	4.50	4.31	8.80	200.00	5.10	6.50	222.40
13	60.00	10.00	3.00	1.22	2.56	56.32	1.46	1.85	64.00
14	55.00	8.00	4.50	2.60	5.28	115.50	3.17	3.82	137.00
15	50.00	6.00	3.00	2.44	4.90	113.00	2.94	3.71	126.00
16	55.00	8.00	4.50	2.65	5.20	124.00	3.09	3.85	129.90
17	55.00	8.00	4.50	2.48	5.40	120.50	3.20	3.70	132.58
18	55.00	8.00	7.00	3.90	8.00	180.36	4.65	5.90	204.80
19	47.00	8.00	4.50	2.92	5.96	136.25	3.50	4.40	152.00

Additionally, repetitions were conducted to calculate the lack of fit, sum of square error, and to validate the precision and accuracy of the previous results. Analysis of variance (ANOVA) was used for graphical analysis of the data, enabling the identification of interactions between the independent input variables and the responses. The response function (*y*) was analyzed using the quadratic Equation (1), where the coefficient of determination (*R* ^2^) was used to assess the model's consistency. Model terms were assessed at a 95% confidence level using *p* values (probability) (Nanvakenari et al. 2021). Three‐dimensional plots were generated for both types of samples (blanched and unblanched) to depict the influence of the aforementioned independent variables.

(1) $$Y=\beta_{0}+\sum_{i=1}^{k}\beta_{i}X_{i}+\sum_{i=1}^{k}\beta_{\mathrm{ii}}X_{i}^{2}+\sum_{i<j}^{k}\beta_{\mathrm{ij}}X_{i}X_{j}$$

### 2.6 Building Artificial Neural Network (ANN)

**FIGURE 3:**
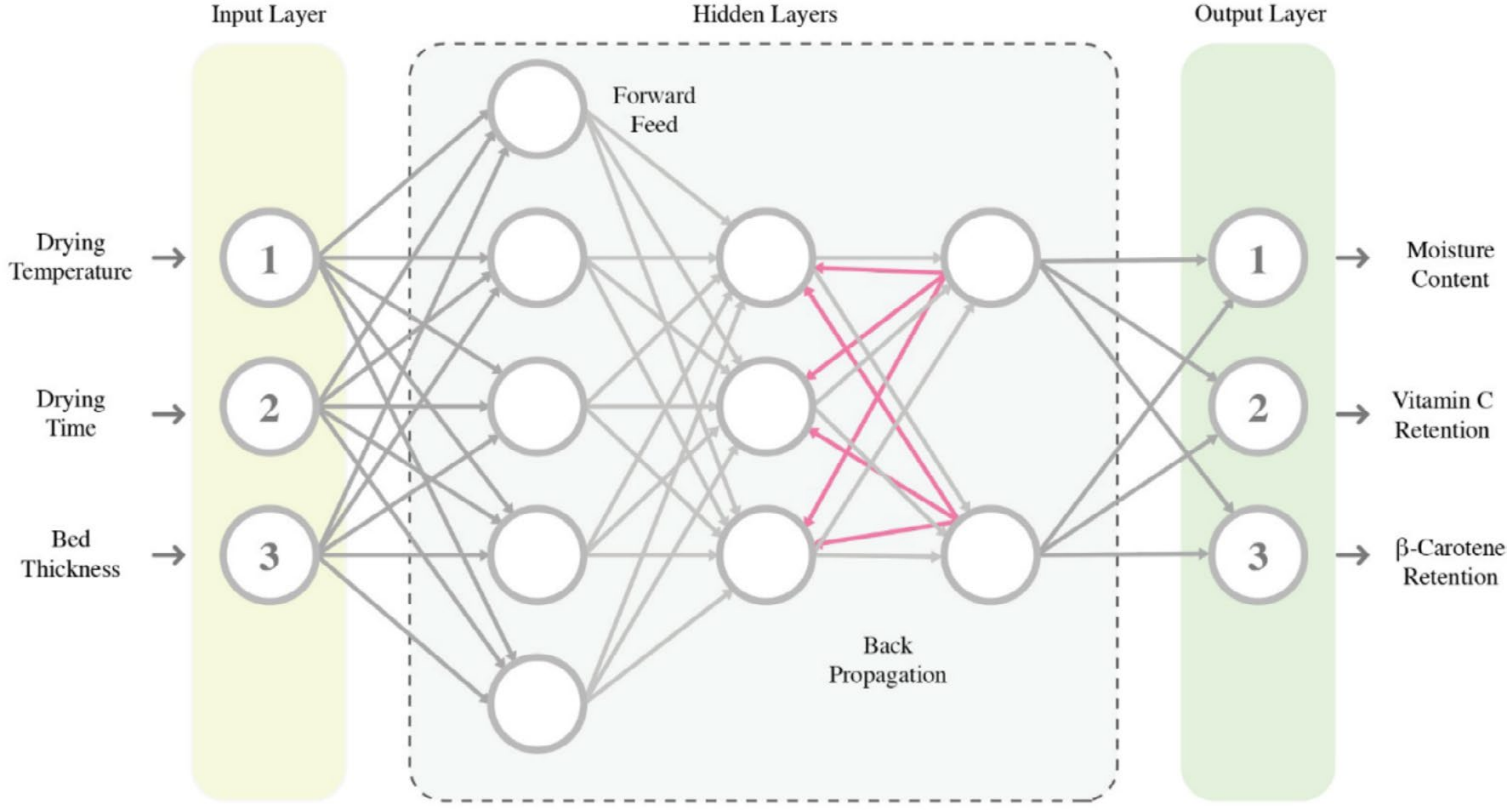
Configuration of the neural network used in the current research.

Artificial neural networks (ANNs) were applied to reproduce the identical experimental data utilized for RSM analysis. A total of 19 patterns were employed in the ANN, each containing six components (X1, X2, X3, Y1, Y2, and Y3), which were divided for training, testing, and validation at a ratio of 70:15:15 accordingly. The input variables consisted of three elements (X1, X2, and X3), while the remaining variables served as outputs. To construct the prediction model, a feed‐forward multilayer perceptron (MLP) artificial neural network trained with a back‐propagation algorithm using MATLAB (version 2021b) was chosen. Figure 3 illustrates the structure of the ANN utilized in this study.

The adjustment of the ANN parameters involved selecting the number of hidden layers and neurons, as well as determining the transfer function type and training function (Figure 1). The ANN model's reliability was evaluated through statistical parameters such as the Average Absolute Relative Deviation percent (AARD%), Mean Square Error (MSE) and correlation coefficient (*R* ^2^). The training was continued until a minimum AARD and MSE with a maximum *R* ^2^ in the validation process were achieved.

### 2.7 Physicochemical Evaluations

Three responses were measured based on the varied input parameters. These responses were analyzed using established calculation and analytical methods. Details regarding the responses, along with other relevant parameters pertaining to the dried water spinach powder, are discussed in the subsequent sections.

#### 2.7.1 Determination of Moisture Content

The moisture content of fresh vegetables was determined following an oven drying method. The fresh sample comprised 5 g was placed into the oven to dry at 105°C to a constant weight. Then the moisture content was determined based on the mass difference between before and after drying the sample following Equation (2) as per the method of AOAC 2000.

(2) $$\%\text{Moisture}=\frac{M_{\text{initial}}-M_{\text{dried}}}{M_{\text{initial}}}\times 100$$

During the drying process, the moisture content was determined as the difference between the initial mass (_initial_) and the mass after drying (_dried_). The basic approach behind this method is the fact that water has a lower boiling point than the other major food components. Therefore, the moisture content after drying by FBD was calculated using Equation (3) described by Hossain et al. (2023).

(3) $$\%\text{Moisture}=\frac{M_{2}}{M_{1}}\;100$$

Where, *M*_2_ and *M*_1_ are the dried sample weight and initial sample weight, respectively.

#### 2.7.2 Estimation of Vitamin C

The estimation of ascorbic acid content was conducted using the method outlined by (Barros et al. 2007; Klein and Perry 1982). A dried methanolic extract (100 mg) was prepared and extracted with 10 mL of 1% metaphosphoric acid at room temperature for 45 min. The extract was then filtered using Whatman No. 4 filter paper. A 1 mL aliquot of the filtrate was mixed with 9 mL of 2,6‐dichlorophenolindophenol, and the absorbance was measured at 515 nm within 30 min of the sample preparation against a blank. The ascorbic acid content was calculated based on a calibration curve of authentic L‐ascorbic acid (0.020–0.12 mg/mL). All assays were performed in triplicate, and the results were reported as mean values ± standard deviations, expressed as mg of ascorbic acid per 100 g of extract and calculated as Equation (4).

(4) $$\text{Amount of ascorbic acid}=\frac{\text{Titre}\;d.f.V\;100}{\text{Aliquot}\;M}$$

Where, *d.f*. means dye factor, *V* means volume made up, and *M* means the weight of the sample.

#### 2.7.3 Estimation of Beta Carotene

To determine the total β‐carotene content, approximately 3.0 g of vegetable leaf powder was weighed in a mortar using a precision balance. Carotenoid extraction was carried out by adding 80% acetone incrementally to form a paste. This paste was then transferred to a sintered funnel (5 μm) connected to a 50 mL Buchner flask and filtered through Whatman paper. The extract was relocated to a 500 mL separatory funnel that contained 20 mL of petroleum ether. The lower layer was discarded, and 2 mL of 5% KOH was added to the remaining extract, which was kept in a dark place for 2 h. After that, 50 mL of double‐distilled water was added to the separatory funnel, and the lower layer was discarded again. The washing process was repeated twice so that no residual acetone remained. Finally, the extract was taken to a volumetric flask of 50 mL by transferring through a funnel, and the absorbance was measured at 453 nm. The β‐carotene amount was estimated using the formula provided by de Carvalho et al. (2012) and stated in Equation (5).

(5) $$\text{Amount of}\;\beta -\text{carotene}=\frac{A\;V10^{4}}{A_{1\mathrm{cm}}^{1\%}\;P}$$

Where *A* denotes absorbance, *V* denotes total extract volume, *P* denotes sample weight, and A1% 1 cm equals to 2592 (β‐carotene Extinction Coefficient in Petroleum Ether).

### 2.8 Predictive Power Comparison

The results obtained from the prediction of RSM and ANN models were subsequently compared with the experimental results from 19 runs developed by Design Expert. Four performance evaluation indexes were compared: Absolute Average Relative Deviation (AARD), Mean Relative Deviation MRD, Mean Squared Error (MSE), and the Coefficient of Determination *R* ^2^. Ideally, the value of *R* ^2^ is close to 1; and for AARD, MRD, and MSE should be minimized. The calculation of these metrics was based on the following established Equations ((6), (7), (8), (9)) as presented by Billah et al. (2024) and Jradi et al. (2022).

(6) $$\%\text{AARD}=\frac{100}{M}\sum_{i=1}^{M}\frac{V_{i}^{\exp}-V_{i}^{\text{pred}}}{V_{i}^{\exp}}$$

(7) $$\mathrm{MSE}=\frac{1}{M}\sum_{i=1}^{M}V_{i}^{\exp}-V_{i}^{\text{pred}}$$

(8) $$\mathrm{MRD}={\left[\frac{1}{M}\sum_{i=1}^{10}{\left(\frac{V_{\text{pred}}-V_{\exp}}{V_{\exp}}\right)}^{2}\right]}^{0.5}100$$

(9) $$r^{2}=\frac{\sum_{i=1}^{M}{\left(V_{i}^{\exp}-\left|V_{i}^{\exp}\right|\right)}^{2}-\sum_{i=1}^{M}{\left(V_{i}^{\exp}-\left|V_{i}^{\text{pred}}\right|\right)}^{2}}{\sum_{i=1}^{M}{\left(V_{i}^{\exp}-\left|V_{i}^{\exp}\right|\right)}^{2}}$$

Here, *exp* means the experimental data and *pred* means the predicted value.

### 2.9 Statistical Analysis

The previously discussed psychochemical analyses were conducted in triplicate, and the obtained data were analyzed using analysis of variance (ANOVA) by statistical software (SPSS windows trial version 22). Duncan's Multiple Range Test was employed to compare group means, ranking them from smallest to largest and calculating a range statistic for each comparison for each analysis. Statistical significance was determined at *p* < 0.05.

## 3 Results and Discussion

### 3.1 Response Surface Model through CCD

The relationship among the three input variables and the three key process responses was assessed in the drying process using RSM. To achieve a well‐fitted model for each response, significant model terms were identified via ANOVA analysis. Central Composite Design (CCD), as shown in Table 2, allowed for the development of mathematical models where the predicted responses (*Y*) were formulated as functions of drying temperature (A), drying time (B), and bed thickness (C). The equations were constructed by combining constant terms with first‐order effects and interaction effects, following Equation (1). Nonsignificant terms were removed after the initial ANOVA analysis, resulting in modified equations. Table 3 provides details of the quadratic models in actual factors and other statistical parameters. Since the system's behavior is complex, evaluating the fitness of the chosen models is necessary. Multiple methods, including residual analysis, lack‐of‐fit testing, and scaling residuals, were employed to confirm model adequacy. The coefficient of determination (*R* ^2^) was used to assess the overall predictive performance of the models. The proximity of this value to one indicates how well the model fits the experimental analysis data. The *R* ^2^ values for the models are presented in Table 3.

**TABLE 3:** Analysis of variance (ANOVA) for response parameters.

Responses	Actual equation	Probability value	Probability of lack of fit (LOF)	*R* ^2^	Adj *R* ^2^	Adequate precision	Standard deviation (S.D)	Coefficient of variance, %
Samples without blanching								
Moisture content (wb) %	Moisture Content (wb) = 5.4735 – 0.0469*A − 0.7296*B + 1.1783*C − 0.0747*B*C + 0.0445*B^2^	< 0.0001	0.3820	0.9911	0.9877	57.4902	0.1139	4.30
Vitamin C, mg/100 g	Vitamin C = 10.0885 – 0.0893*A − 2.1026*B + 3.9292*C + 0.0140*A*B − 0.02680*A*C − 0.0159*B*C + 0.0836*B^2^	< 0.0001	0.1408	0.9958	0.9931	69.4597	0.1738	3.21
β‐carotene, μg/g	β‐carotene = 231.8734 – 2.0223*A − 30.7449*B + 56.2976*C − 3.6588*B*C + 1.9282*B^2^	< 0.0001	0.3887	0.9903	0.9866	55.0070	5.51	4.40
Samples with blanching								
Moisture content %	Moisture Content (wb) = 6.0498 – 0.0573*A − 0.7406*B + 1.4425*C − 0.0933*B*C + 0.0462*B^2^	< 0.0001	0.1140	0.9918	0.9886	59.8199	0.1307	4.09
Vitamin C, mg/100 g	Vitamin C = 8.4310 – 0.0705*A − 1.1532*B + 1.7838*C − 0.1132*B*C + 0.0705*B^2^	< 0.0001	0.0749	0.9924	0.9895	62.4578	0.1589	3.98
β‐carotene, μg/g	β‐carotene = 114.6338 – 0.3477*A − 33.8888*B + 95.3680*C − 0.6105*A*C − 3.9322*B*C + 2.0897*B^2^	< 0.0001	0.4363	0.9950	0.9925	70.0713	4.59	3.30

The ANOVA table presents the *p* values to be found to be < 0.05 for all models, which suggests that the models are significant at the 95% confidence level. As a diagnostic tool, lack of fit (LOF) was tested to compare pure error and evaluate model adequacy based on replicated measurements. If the LOF is found to be significant, it means the model fails to accurately predict the output. In Table 3, it is shown that there are high *p* values for lack of fit that indicate strong model correlation between input variables and responses (Behera et al. 2018; Pilkington et al. 2014).

Another crucial parameter, adequate precision (AP), was used to compare the range of predictions to the average prediction error. An AP ratio greater than 4 indicates good model discrimination (Movagharnejad et al. 2019). In Table 3, all the models display an AP greater than 4, confirming their ability to navigate the design space defined by CCD. The coefficient of variation (CV) assesses model reproducibility, and values below 10% are generally accepted as reproducible (Bagherlou et al. 2024). All responses exhibit CV values under 10%, suggesting good reproducibility. The standard deviation (S.D.) measures the error in the design and is expected to be small (Nanvakenari et al. 2021). In our study, the standard deviations for moisture content and amount of vitamin C were relatively low for both the blanched and unblanched samples. However, the β‐carotene response exhibited a higher standard deviation. This discrepancy could be attributed to measurement at the micro‐unit level and the possibility of unexpected errors during estimation.

**FIGURE 4:**
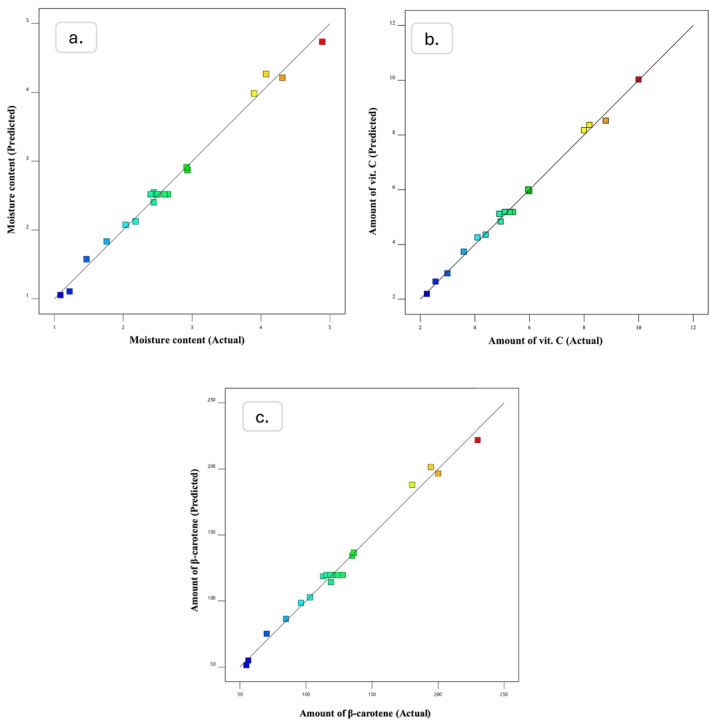
Predicted versus actual values plots produced by Design expert; (a) Moisture content, (b) amount of Vit. C, and (c) amount of ß‐carotene for unblanched samples.

**FIGURE 5:**
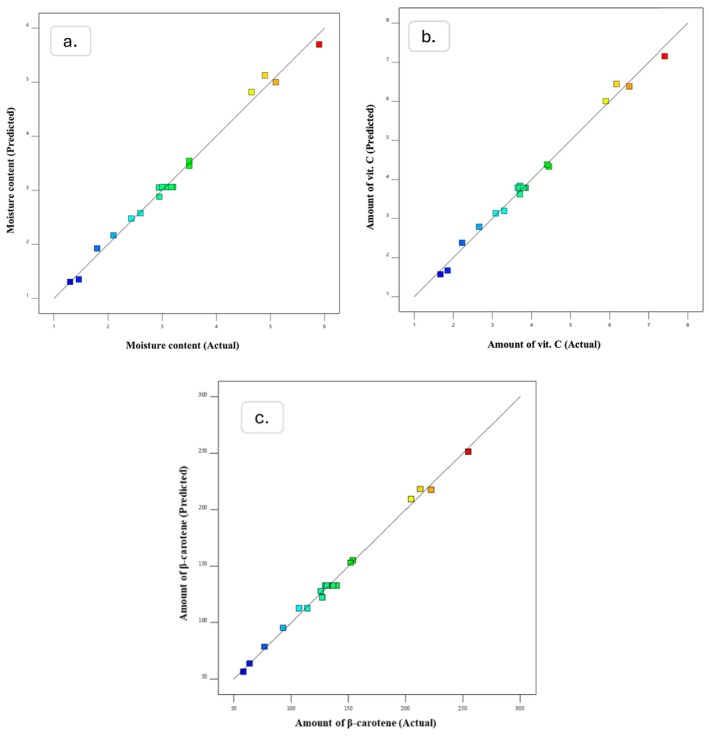
Predicted versus actual values plots produced by Design expert; (a) Moisture content, (b) amount of Vit. C, and (c) amount of ß‐carotene for blanched samples.

Finally, model reliability can be visually validated by diagnostic plotted graphs, for example, predicted versus actual diagrams. Figures 4 and 5 illustrate these plots for the unblanched and blanched samples, indicating a strong relationship between experimental and predicted data.

### 3.2 Analysis of Responses

The lab‐scale FBD was run according to the conditions suggested by the experimental design. For each run, three responses were recorded as per analytical standards and the statistical approach.

#### 3.2.1 Moisture Content Attainment

**FIGURE 6:**
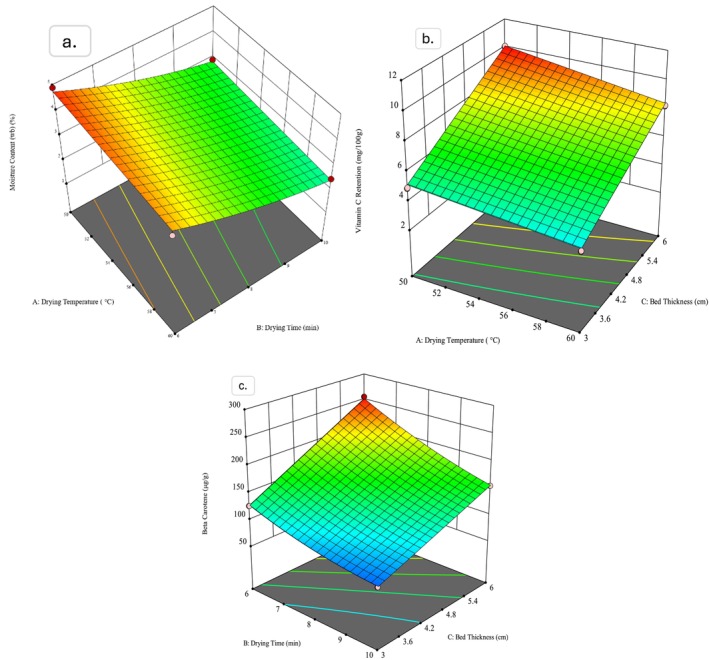
Response surface plot produced by Design expert; (a) Moisture content (b) amount of Vit. C, and (c) amount of ß‐carotene for unblanched samples.

**FIGURE 7:**
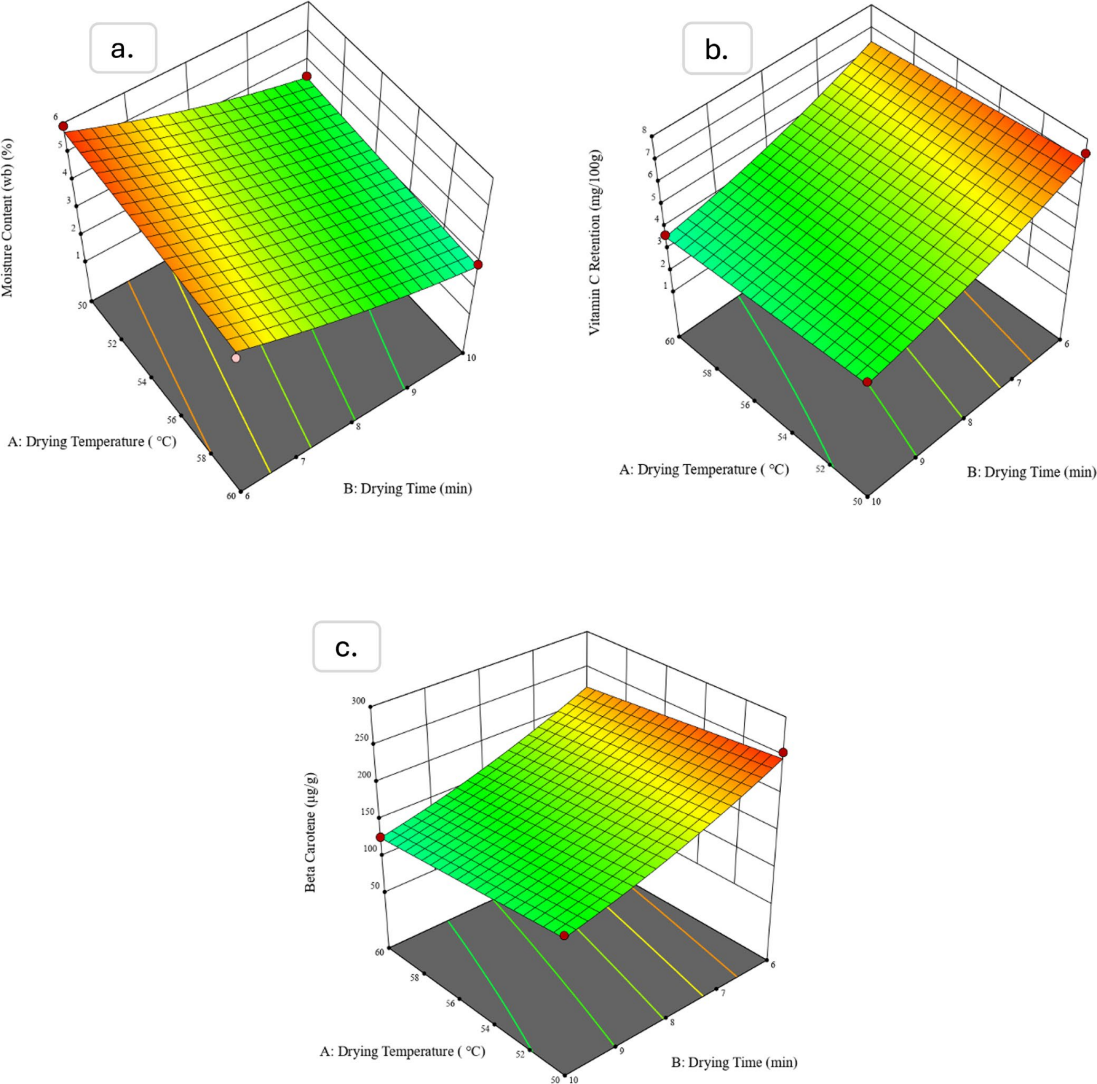
Response surface plot produced by Design expert; (a) Moisture content (b) amount of Vit. C, and (c) amount of ß‐carotene for blanched samples.

The attainment of target moisture content is a critical response in every drying operation, as it directly affects the quality and shelf life of the final product. Figures 6a and 7a illustrate the simultaneous effect of drying temperature, drying time, and bed thickness on the moisture content of dried water spinach for unblanched and blanched samples, respectively. The figures clearly demonstrate that bed thickness exerts a prevailing influence on moisture content compared to the other two factors, drying temperature and drying time. However, the direct impact of drying temperature and drying time is also evident and is further supported by the coded equations obtained from ANOVA.

The coded equations of unblanched and blanched samples show the calculated coefficients as +1.1783, −0.0469, −0.7296; and + 1.4425, −0.0573, −0.7406 for bed thickness, drying temperature, and drying time, respectively. The coefficients indicate that both the drying time and drying temperature exert a strong negative influence on moisture content for both types of samples, which implies that increasing the drying time and temperature leads to a significant reduction in moisture content (Billah et al. 2024; Chua et al. 2003). This observation aligns with the practical drying behavior as particles are exposed to hot air circulation for extended periods; internal moisture migrates to the surface and quickly restores the moisture gradient until the equilibrium moisture content (EMC) is achieved (Akhtaruzzaman et al. 2021; Sarker et al. 2015).

Again, the negative coefficient of drying temperature indicates an inverse relationship with the attainment of moisture. As the drying temperature increases, moisture vaporization within the vegetable particles is accelerated by heat energy that leads to the faster removal of moisture (Nazghelichi et al. 2010; Thibault et al. 2024). This effect is particularly evident in both blanched and unblanched samples, which show similar behavior in reducing moisture content. However, the influence of bed thickness supersedes the other two input parameters, especially for the case of blanched samples, where the effect is more evident.

Overall, the findings highlight the importance of optimizing bed thickness in drying operations to ensure efficient removal of moisture where drying time and temperature play secondary but still significant roles. The differences observed between blanched and unblanched samples also focus on the role of blanching in modifying the drying kinetics and response to process variables.

#### 3.2.2 Vitamin C Estimation

Water spinach is a valuable source of various vitamins, particularly vitamin C, which is abundant in this vegetable. Therefore, the retention of vitamin C was analyzed as one of the key response variables across the RSM runs. Figures 6b and 7b illustrate the simultaneous effects of drying temperature, drying time, and bed thickness on the vitamin C content of dried spinach for unblanched and blanched samples, respectively. The coded equations derived from the analysis of variance (ANOVA) provide insight into the relationships between these input variables and the vitamin C content.

The intercept values in the coded equations are +10.0885 and + 8.4310 for unblanched and blanched samples, respectively. These intercepts indicate that if the other factors are held at zero, the estimated amount of vitamin C in the dried spinach would be 10.09 mg/100 g for unblanched samples and 8.43 mg/100 g for blanched samples. The lower intercept value for blanched samples reflects the degradation of ascorbic acid due to exposure to heat, immersion in hot water, and light during the blanching process, which is consistent with the known susceptibility of ascorbic acid to these conditions (Joseph Bassey et al. 2024).

The coefficient values for the input variables mentioned as drying temperature (DT), drying time (Dt), and bed thickness (Bt) are −0.0893, −2.1026, +3.9292; and − 0.0705, −1.1532, +1.7838 for unblanched and blanched samples, correspondingly. Based on the values, it is observed that bed thickness employs the most significant positive influence on vitamin C retention for both samples, while drying time holds a significant negative impact. The negative coefficient for drying temperature, which is comparatively lower than others, indicates a less but still detrimental effect on vitamin C content, particularly in unblanched samples.

The marked negative impact of drying time on vitamin C retention could be attributed due to prolonged exposure to the drying environment, which results in an acceleration of ascorbic acid degradation (Boateng 2024). This effect is more distinguishable in unblanched samples, which showed greater sensitivity to the drying process compared to blanched samples. The findings of the current study suggest that blanching could mitigate some vitamin C loss by deactivating oxidative enzymes; however, the overall retention of vitamin C is still greatly influenced by the drying parameters, particularly bed thickness and drying time.

#### 3.2.3 ß‐Carotene Estimation

The response surface plots of the combined influence of input parameters on beta‐carotene retention are illustrated in Figures 6c and 7c. The intercept values retained from ANOVA, which are calculated as +231.8734 and + 114.6338 for unblanched and blanched samples, respectively, indicate that blanched samples preserved more beta‐carotene. The reason behind the higher retention could be due to blanching as a pretreatment, which inactivates enzymes like polyphenol oxidase and lipoxygenase that degrade beta‐carotene during drying (Moura et al. 2024). Additionally, cell walls are softened by the blanching process, which ensures uniformity in drying and reduces the risk of beta‐carotene degradation. The three coefficients DT, Dt, and Bt valued according to ANOVA are −2.0223, −30.7449, +56.2976; and − 0.3477, −33.8888, +95.3680 for unblanched and blanched samples accordingly. Like the previously discussed responses, the coefficient values show that bed thickness possesses the most significant positive influence on beta‐carotene retention and drying time as the second most important influencing factor. Although drying temperature negatively affects beta‐carotene retention, the impact is less because of the lowered coefficient value compared to the other two input variables. Overall, both sample types reveal similar effects on beta‐carotene retention, where blanched samples present to have an advantage due to their antioxidative properties and reduced degradation during the drying process.

#### 3.2.4 Optimization of the Drying Process

**FIGURE 8:**
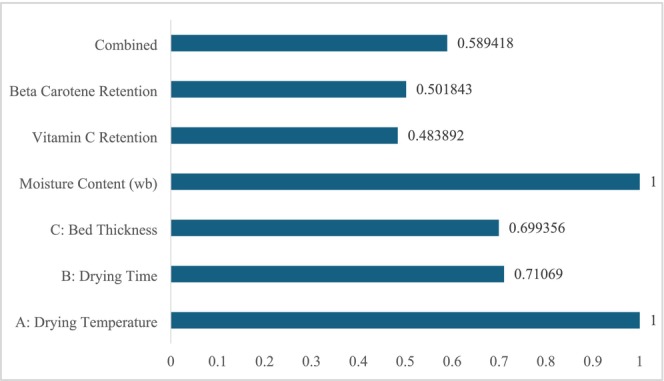
Input factors, responses, and combined optimized desirability values.

A RSM module was employed to identify the optimal combination of factor levels, which aligned with the predefined criteria for every response along with each factor simultaneously. The desired intentions were set to maximize the vitamin C and ß‐carotene content, while minimizing the moisture content. The independent variables, specifically drying temperature, drying time, and bed thickness, were kept within a specified range. The multiobjective optimization process assigned the same weights to each response. As the two sample types were run separately, the Design Expert software integrated defined goals into two separate general objective functions, ultimately leading to two optimized conditions with a combined desirability score for the unblanched and blanched samples, respectively. By comparing the two optimized conditions, it was found that the unblanched sample suggested a desirability value of 0.589, as illustrated in Figure 8. The desirability function serves as an objective measure that aids in the optimization of parameters (Behera et al. 2018; Movagharnejad et al. 2019). The desirability function was quantified as 1, 0.7107, and 0.6993 for the input parameters: drying temperature, drying time, and bed thickness. The predicted optimal values for both dependent and independent variables are listed in Table 4.

**TABLE 4:** Optimal point selection in the desirability regime.

Drying parameters	Target	Drying temperature, °C	Drying time, min	Bed thickness, cm	Sample type	Moisture content (wb), %	Vitamin C, mg/100 g	Beta‐carotene, μg/g
Drying temperature (DT), °C	Is in range	60.00	7.16	5.10	Unblanched	3.00	—	—
Drying time (Dt), min	Minimize	60.00	7.16	5.10	Unblanched	—	5.99	—
Bed thickness (Bt), cm	Maximize	60.00	7.16	5.10	Unblanched	—	—	142.73
DT, Dt, Bt	Range, min, max	60.00	7.45	4.47	Blanched	3.00	—	—
		60.00	7.51	4.51	Blanched	—	3.73	—
		60.00	7.51	4.51	Blanched	—	—	130.71
Optimal point		60.00	7.16	5.10	Unblanched	3.00	5.99	142.73

The optimal conditions were identified at a drying temperature of 60°C, a drying time of 7.16 min, and a bed thickness of 5.1 cm to dry unblanched samples. Under these optimized settings, the predicted outcomes for moisture content, vitamin C, and ß‐carotene were 3.00%, 5.99 mg/100 g, and 142.73 μg/g, respectively. To validate the predicted optimized conditions, an experimental run was performed, and the results, along with the predicted values, are detailed in Table 5. The errors calculated between the predicted and experimental values were found to be minimal (< 5%), with deviations of 1.00%, 2.50%, and 4.36% for moisture content, vitamin C amount, and ß‐carotene amount, respectively (Sun 2022; Van Hooren et al. 2024). These results indicate a high level of agreement between the experimental and predicted values, confirming the accuracy of the optimization process.

**TABLE 5:** Verification of the drying process at optimum conditions.

	Drying temperature, °C	Drying time, min	Bed thickness, cm	Type of sample	Moisture content (wb), %	Vitamin C, mg/100 g	Beta‐carotene, μg/g	Desirability
RSM prediction	60.00	7.16	5.10	Unblanched	3.00	5.99	142.73	0.589
Experimental observation	60.00	7.16	5.10	Unblanched	2.97	5.84	136.50	—
Error, %	—	—	—	—	1.00	2.50	4.36	—

### 3.3 ANN Modeling

The initial step in Artificial Neural Network (ANN) modeling involved optimizing the network to minimize dimensionality and reduce both training and testing errors. A close agreement between the predicted and experimental data is indicated when a minimal MSE and AARD values, along with a maximum *R* ^2^ is achieved. Multiple architectures of network were evaluated to identify the most precise configuration. A continued trial‐and‐error process led to the determination of the optimal number of neurons. The final selected model employed a back‐propagation neural network where two hidden layers were chosen. Again, the model engaged the Levenberg–Marquardt (LM) training algorithm along with a logsig–logsig–purelin function for transferring, a similar application demonstrated by Nanvakenari et al. (2021). The optimal configuration for hidden layer neurons was found to be 2‐8‐7‐5.

The ANN model was subsequently used to predict six responses from unseen data (for blanched and unblanched samples) to assess the network's predictive accuracy. The prediction details for all the runs are enlisted in Table 6. From the analysis of unseen data, it was evident that the network possessed satisfactory predictive power, indicating strong compatibility between the predicted and experimental results.

**TABLE 6:** Results of RSM and ANN prediction.

No.	Input			Samples without blanching						Samples with blanching					
	Drying temperature, °C	Drying time, min	Bed thickness, cm	Prediction by RSM			Prediction by ANN			Prediction by RSM			Prediction by ANN		
				Moisture content (wb), %	Vitamin C, mg/100 g	Beta‐Carotene, ug/g	Moisture content (wb), %	Vitamin C, mg/100 g	Beta‐Carotene, ug/g	Moisture content (wb), %	Vitamin C, mg/100 g	Beta‐Carotene, ug/g	Moisture content (wb), %	Vitamin C, mg/100 g	Beta‐Carotene, ug/g
1	60.00	6.00	3.00	2.09	4.26	97.73	2.03	4.12	96.37	2.48	3.13	112.84	2.45	3.58	115.39
2	63.00	8.00	4.50	2.15	4.35	102.56	2.19	4.38	102.92	2.58	3.2	112.72	2.48	3.40	118.33
3	55.00	8.00	4.50	2.53	5.18	121.26	2.53	5.29	121.95	3.06	3.79	132.9	2.97	4.06	133.78
4	50.00	6.00	6.00	2.53	5.18	121.26	2.53	5.29	121.95	3.06	3.79	132.9	5.92	7.40	255.02
5	50.00	10.00	3.00	4.87	10.02	228.57	4.22	9.39	224.74	5.7	7.15	251.43	1.49	2.09	67.68
6	50.00	10.00	6.00	1.43	2.94	69.27	1.41	2.99	67.01	1.93	2.38	78.67	3.23	4.08	153.22
7	60.00	6.00	6.00	2.89	5.94	133.61	2.92	5.99	135.04	3.45	4.33	155.24	4.71	6.77	218.85
8	60.00	10.00	6.00	4.12	8.36	195.56	4.05	8.16	193.87	5.13	6.45	218.28	2.76	3.61	126.18
9	55.00	11.36	4.50	2.38	4.84	113.75	2.51	5.04	115.62	2.88	3.63	122.09	3.17	1.38	4.50
10	55.00	8.00	1.98	1.84	3.73	87.37	1.76	3.61	84.90	2.17	2.79	95.5	0.81	2.07	55.73
11	55.00	4.64	4.50	1.02	2.19	49.39	1.51	10.44	57.78	1.31	1.57	56.37	3.83	8.63	236.28
12	60.00	10.00	3.00	4.22	8.52	197.41	4.30	2.56	204.51	5	6.39	217.58	1.56	2.37	76.48
13	50.00	6.00	3.00	1.25	2.64	55.83	1.22	4.94	56.35	1.35	1.67	63.84	2.94	3.47	122.88
14	55.00	8.00	7.02	2.53	5.18	121.26	2.53	7.99	121.95	3.06	3.79	132.9	4.62	5.59	200.74
15	47.00	8.00	4.50	2.52	5.11	114.32	2.45	6.14	112.94	3.05	3.84	127.67	3.59	4.71	158.43

### 3.4 Predictive Power Comparison Between RSM and ANN Models

To investigate the predictive competences, the RSM and ANN models were competed. The comparison was based on four key performance indicators such as AARD, MRD, MSE, and *R* ^2^. Table 6 presents the predicted values for both models. The objective of this comparison was to achieve and observe minimized values for AARD, MRD, and MSE while a maximized value for *R* ^2^ approached close to 1. The comparative parameters that are calculated based on the data presented in Table 6 show excellent prediction as compared with the experimental data suggested by similar works (Billah et al. 2024; Chokphoemphun et al. 2024; Chuwattanakul et al. 2022; Nanvakenari et al. 2021; Pilkington et al. 2014; Sarker et al. 2015). Regarding all the outputs, both RSM and ANN models showed strong adaptability to experimental data with high accuracy; however, the ANN model demonstrated more deviation compared to RSM in most cases. The values of performance indicators are detailed in Table 7. Overall, RSM showed a significantly higher potential for generalization than the ANN model.

**TABLE 7:** Predictive power comparison of Response surface methodology and artificial neural network for three outputs.

Parameters	Moisture content (wb), %		Vitamin C, mg/100 g		Beta‐carotene, μg/g	
	RSM	ANN	RSM	ANN	RSM	ANN
AARD, %	2.5443	4.0950	2.2191	2.5188	1.8168	1.4264
MRD	3.0013	9.6263	2.5230	4.9788	2.8773	2.1294
MSE	0.0043	0.0354	0.0175	0.1670	5.4221	5.5890
*R* ^2^	0.9957	0.9728	0.9957	0.9651	0.9976	0.9974

## 4 Conclusions

In this research, the influence of key drying parameters, namely drying temperature, drying time, and bed thickness, on the drying performance of water spinach using a fluidized bed dryer was evaluated. Two types of samples, such as blanched and unblanched samples, were investigated in the study. The findings have demonstrated that all the considered variables significantly affect the responses (moisture content, amount of vitamin C and beta‐carotene). The study has revealed that the samples that underwent blanching show a greater effect on moisture retention in response to the change in bed thickness, quantified as 16% higher response than the unblanched samples. Conversely, the samples that did not undergo the pretreatment stage have showed a 25% higher response in vitamin C retention during the change in bed thickness than the blanched samples. In terms of beta‐carotene content, a minimal variance in response has been observed between the two treatments. To conduct the comparison between the predictive capabilities of RSM and ANN, four performance indicators were taken into consideration, such as AARD, MRD, MSE, and *R* ^2^. After analyzing the performance indicators, it has been proven that the RSM model has performed better for almost all the cases in terms of precision and generating predictive results close to the experimental data. The RSM model has also patched a quadratic model that provides adequate mathematical details of the fluidized bed drying process by prescribing the optimized process parameters. The optimal drying conditions suggested by the response surface methodology comprise a drying temperature of 60°C, a drying time of 7.16 min, and a bed thickness of 5.10 cm through the desirability function (*D* = 0.589) for the unblanched samples. Under these optimal conditions, the conducted experimental tests yielded results of 2.97% moisture content, 5.84 mg/100 g vitamin C, and 136.50 μg/g beta‐carotene. The close alignment between experimental and predicted outcomes confirms the robustness of the selected models, emphasizing their potential for application in industrial‐scale drying of leafy vegetables like water spinach.

## Author Contributions

**Mir Tuhin Billah:** conceptualization (equal), formal analysis (equal), methodology (equal), supervision (equal), validation (equal), writing – original draft (equal). **Noor E Zannat:** formal analysis (equal), software (equal), validation (equal), writing – review and editing (equal). **Md Akram Hossain:** formal analysis (equal), visualization (equal), writing – review and editing (equal). **Ishmam Haque Sachcha:** visualization (equal), writing – review and editing (equal). **Sabina Yasmin:** formal analysis (equal), validation (equal). **Md. Sazzat Hossain Sarker:** methodology (equal), supervision (equal), writing – review and editing (equal).

## Conflicts of Interest

The authors declare no conflicts of interest.

## Data Availability

The data that support the findings of this study are available from the corresponding author upon reasonable request.
